# Perceived Symptoms, Mental Health and Quality of Life after Hospitalization in COVID-19 Patients

**DOI:** 10.3390/jpm12050728

**Published:** 2022-04-30

**Authors:** Evangelos C. Fradelos, Stylianos Boutlas, Eleni Tsimitrea, Alexandra Sistou, Konstantinos Tourlakopoulos, Ioanna V. Papathanasiou, Konstantinos I. Gourgoulianis

**Affiliations:** 1 Department of Nursing, University of Thessaly, 41500 Larissa, Greece; e79tsi@yahoo.gr (E.T.); iopapathanasiou@yahoo.gr (I.V.P.); 2 Department of Respiratory Medicine, Faculty of Medicine, University of Thessaly, 41110 Larissa, Greece; sboutlas@gmail.com (S.B.); alexiasist@gmail.com (A.S.); kntourlakopoulos@gmail.com (K.T.); kgourg@uth.gr (K.I.G.)

**Keywords:** COVID-19, mental health, quality of life, symptoms

## Abstract

Patients recovering from novel coronavirus are reporting a variety of symptoms such as cough, dyspnea, myalgia as well as psychological distress and poor quality of life. The aim of this study is to assess quality of life and psychological distress in COVID-19 survivors and the sociodemographic and clinical characteristics that affect COVID-19 survivors’ mental health status and quality of life. A quantitative study was conducted among COVID-19 survivors, who had previously been admitted to the University Hospital of Larissa, Greece. Data were collected via a questionnaire consisting of three-parts. The first part consisted of questions about the demographic characteristics. The second part was the SF-36 QoL index. The third part was the Symptom Checklist-90r (SCL 90-R). In addition, clinical information such as the length and the department of hospitalization, days since discharge and pulmonary function (spirometry values) were recorded. From a total of 145 patients, 60% were male, aged 59.72 ± 12.74 and 78.6% of them were married; the majority had completed secondary education, 35.9% were pensioners and 58.6 were living in urban areas. The most frequently reported symptoms were fatigue (67.6%) and pain (44.8%) and 11.7% were experiencing psychological distress. Pain, loss of smell, mandatory education, ICU admission, female gender and the experiencing of skin disturbance are associated with poor physical QoL among COVID-19 recovered patients. Greek COVID-19 previously hospitalized patients were reporting several symptoms associated with COVID-19. Good QoL and mental health were also reported. Physical pain, loss of smell and female gender were associated with poor QoL and psychological distress.

## 1. Introduction

In December 2019, a pneumonia outbreak in Wuhan, China, was reported, with symptoms including fever, dry cough, exhaustion, and gastrointestinal symptoms. The pandemic pathogen was identified as a novel coronavirus, named the novel coronavirus 2019 (2019-nCoV) [1]. A history of fever (68.7% of patients), cough (68.5%), and/or dyspnea (65.8%) were the three most common symptoms upon admission, according to the latest report from the International Severe Acute Respiratory and Emerging Infection Consortium [2]. Overall, 7.5% of the cases diagnosed between August 2020 and May 2021 and reported by EU/EEA countries to the European System of Supervision (TESS) were hospitalized, although this varied with age. More than two weeks following the confirmed diagnosis of SARS-CoV-2, up to 80% of people had reported at least one symptom [3].

However, in addition to symptoms, clinical manifestations, and survival rates, researchers are increasingly focusing on patients’ quality of life (QoL) and mental health as indicators of illness prognosis [4]. Some studies have been performed to evaluate the quality of life in COVID-19 patients and survivors. COVID-19, according to researchers, can cause physical and psychological impairment, as well as a reduction in health-related quality of life (HRQoL) [5,6].

According to a recent study conducted in Ethiopia, COVID-19 had a significant negative impact on patients’ HRQoL, particularly among the elderly and those with comorbidities [7]. One systematic review, which aimed to assess how COVID-19 affected patients’ quality of life (QoL) after discharge or recovery, found that regardless of the time since discharge or recuperation, the QoL of post-COVID-19 patients was significantly impacted. Female sex, advanced age, the existence of co-morbidities, ICU admission, extended ICU stay, and mechanical ventilation were the most common factors linked with a low level of QoL. Physical role and physical function were found to be the most and least affected domains of QoL in studies conducted using the SF-36, respectively [8]. Another study which aimed to assess COVID-19’s influence on the quality of life (QoL) of survivors, their partners, and family members, concluded that the virus had a long-term negative influence on COVID-19 survivors physical and mental health. Pain and discomfort were the most frequently reported impact, followed by usual activities and anxiety and depression. Hospitalized survivors and survivors with pre-existing health conditions reported a lot more difficulties with mobility and daily activities, as well as having a greater impact on self-care [9].

The COVID-19 epidemic impacted not just QoL, but also the mental health status of COVID-19 survivors. A recent study discovered that around 10% of COVID-19 survivors had anxiety or depression, and that a large proportion (29.5 percent) of survivors had sleeping difficulties. Post-discharge residual symptoms, worry about recurrence, and worry about infecting others were all linked to anxiety and depression [10]. Similar were the findings of another follow-up study, according to which COVID-19 survivors had a significantly higher risk of depression and anxiety than the control group (relative risk, RR = 1.2, 95 percent CI: 1.11.4, *p* = 0.002; and RR = 1.4, 95 percent CI: 1.21.7, *p* = 0.001, respectively). In this study, 14.2% of the participant survivors were found to have clinically defined depression and 12.2% were found to have clinically defined anxiety. Retesting positive for SARS-CoV-2, living alone, female gender, concomitant chronic physical conditions, and poor education and income levels were all risk factors for more severe mental health problems [11]. In the twelve months following infection, pathological fatigue has also been detected [12]. Moreover, in a study with the participation of ten patients who recovered from COVID-19 pneumonia without complications, 10% of them experienced depression and posttraumatic stress disorder one month after discharge, while half of them had depression during treatment. The intensity of posttraumatic stress disorder symptoms was affected by perceived stigma and a history of psychiatric treatment. Survivors also expressed fear about infecting others and about being discriminated against, and stated that following discharge, they opted to avoid contact with others [13].

According to the WHO International Classification of Functioning, Disability, and Health (ICF) framework, rehabilitation programs should be designed in order to promote individual functioning at three levels, including physical function and structure, activity and participation, as well as environmental and personal factors to enhance quality of life. The ICF framework should be used in the areas of evaluation, diagnosis, and interventions, according to current standards. As a result, documenting post-COVID-19 physical and mental health repercussions that affect quality of life is critical in order to improve rehabilitation services and assist healthcare institutions in planning effective rehabilitation programs [14].

Thus, the aim of the present study is to identify mental health status and QoL after being admitted for COVID-19 to a hospital. Furthermore, our aim is also to search which sociodemographic and clinical characteristics affect COVID-19 survivors’ mental health status and quality of life.

## 2. Materials and Methods

A quantitative study was conducted among COVID-19 survivors, who had previously been admitted to the University Hospital of Larissa, Greece. Convenience sampling was used to recruit participants from patients who were recruited during the initial follow-up after discharge. A total of 145 patients were included.

The criteria for inclusion were as follows: patients no longer required oxygen; body temperature was within the normal range for at least 48 without the use of any medicine. The exclusion criteria were: patients who had been diagnosed with a mental disease in the past; patients who have been diagnosed with a terminal illness or who are in the advanced stages of cancer; patients suffering from cognitive impairments.

### 2.1. Data Collection

#### Instrument

A three-part questionnaire was used.

The first part consisted of questions about the demographic characteristics of the patients, such as age, sex, marital status, level of education, employment status as well as questions regarding self-reported symptoms.

The second part was the SF-36 QoL index. SF-36 is a very popular scale that evaluates health-related quality of life [15,16], and its reliability and validity have been documented [17]. The Short Form 36 (SF-36) questionnaire has previously been validated for the Greek population. On the basis of gender, age, and socioeconomic status, it was determined to have good internal consistency, reliability, convergent and discriminative validity, and the ability to distinguish between groups of respondents in the predicted manner (known-groups validity) [16,17]. The multidimensional structure of the Greek version of the instrument has also been confirmed [17,18]. SF-36 consists of eight subscales: vitality (VT), physical functioning (PF), bodily pain (BP), general health perceptions (GH), physical role functioning (RP), emotional role functioning (RE), social role functioning (SF), and mental health (MH). SF-36 also provides two summary scores for QoL, the physical component summary and the mental component summary. Each of the subscales is rated between 0 and 100 with the higher scores indicating better health status. A mean score of 50 has been considered a normative value for all subscales [15,16].

The third part was the Symptom Checklist-90r (SCL 90-R). SCL-90r is a valid and widely used self-completed scale of measurement that examines the subjective discomfort and symptomatic behavior in various dimensions of psychopathology. It consists of 90 questions in a 5-point Likert type (0 = none to 4 = too much) and assesses the nine major symptomatological dimensions—subscales: Somatization (12 items), Obsessive Compulsive (10 items), Interpersonal Sensitivity (9 items), Depression (13 items), Anxiety (10 items), Hostility (6 items), Phobic Anxiety (7 items), Paranoid Ideation (6 items), and Psychoticism (10 items). It also includes seven questions that are not included in the above dimensions and are related to sleep and food disorders (they are referred to in the text as “Additional Items” and are counted in the total score). In addition to the nine subscales, it provides three total psychopathology indicators: (a) Global Severity Index (GSI), (b) Positive Symptoms Total (PST), and (c) Positive Symptom Distress Index (PSDI). The scale has been standardized in the Greek population and has been found to have satisfying criterion validity and sufficient convergent validity [19].

In addition, clinical information such as the length and the department of hospitalization and days since discharge were recorded. Upon their follow-up, pulmonary function (spirometry values) Forced Vital Capacity (FVC), Forced Expiratory Volume (FEV1), FEV1/FVC, Diffusing Capacity of The Lungs For Carbon Monoxide (DLCOc) lit were also recorded.

### 2.2. Ethical Issues

The study was approved by the Institutional Review Board (IRB)/Ethics Committee (EC) of the University Hospital of Larissa (IRB/EC approval reference number: 15314/21-04-2021). The research was carried out in accordance with the Helsinki declaration. Furthermore, all participants gave written informed consent and were informed that they could leave the research at any moment.

### 2.3. Statistical Analysis

The research statistics of the empirical data were processed with SPSS v. 22.0 for Windows (SPSS, Inc., Chicago, IL, USA). Descriptive and inferential statistical methods were generated. Continuous variables are presented with the mean, standard deviation, median, interquartile range, and range (min–max), while categorical variables are presented as absolute (*n*) and relative (%) frequencies. The outcomes (dependent variables) were SF-36 summary scores and the General severity index (GSI) of the Symptom Checklist 90-R scale, while the determinants (independent variables) included patient characteristics. Initially, a bivariate analysis was performed. Student’s t-test and one-way analysis of variance were used for the association between categorical and continuous variables, and Pearson’s correlation coefficient for the correlation between continuous variables. Multivariate linear regression analysis (stepwise method) was applied to identify predictors of summary scores of SF-36 and general severity index (GSI) of Symptom Checklist 90-R scale. Regression coefficients (β) with standard errors and 95% confidence intervals were computed. All reported *p*-values were two-tailed, and the statistical significance level was set at 0.05.

## 3. Results

From a total of 145 patients, 60% were male, aged 59.72 ± 12.74. Furthermore, 78.6% were married, the majority had completed secondary education, 35.9% were receiving pensions, and 58.6% were living in urban areas. Detailed sociodemographic information is presented in Table 1.

Regarding the clinical profile of the patients, 43.4% of the participants were diagnosed with two or more chronic conditions, 9% were treated in the ICU and the mean length of hospitalization was 11.2 days. Detailed information regarding the clinical characteristics is presented in Table 1.

**Figure 1:**
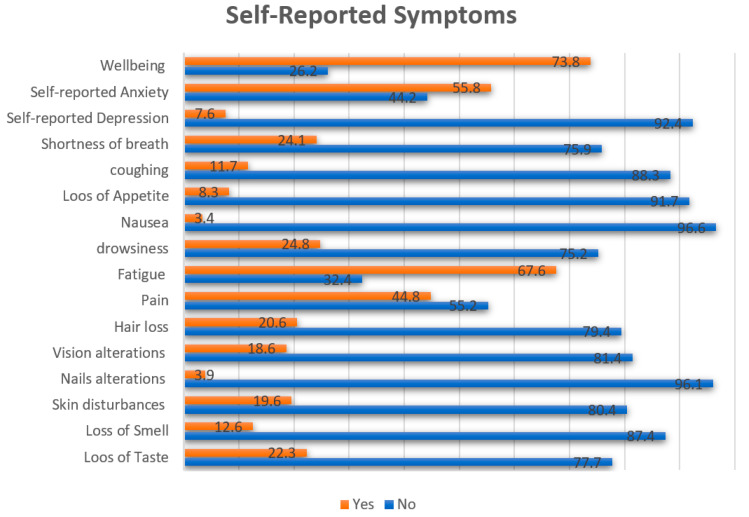
Self-reported symptoms. Values are presented in percentages.

The most frequently reported symptoms were fatigue (67.6%) and pain (44.8%), while the least frequent symptoms were nausea (3.4%) and loss of appetite (8.3%). Detailed information regarding the self-reported symptoms is presented in Figure 1.

Concerning the spirometry lung function values, the mean value of FVC was 3.40 ± 0.92, FEV1 2.76 ± 0.81 and DLCOc 6.60 ± 1.80. Detailed results of lung function are presented in Table 2.

Regarding psychological distress among COVID-19 survivors, the mean value of the general symptom index was found to be 0.44; while, according to cut-off values, 11.7% were experiencing psychological distress. For the subdomain scores, somatization and obsessive-compulsive symptoms were most frequently reported, followed by depressive symptoms. Detailed information regarding psychological distress is presented in Table 3.

The descriptive results for the scores in the SF-36 are presented in Table 4. Both the physical health component summary and the mental health component summary were higher than 50, which shows that patients experience good QoL. Moreover, the mental health component summary scored higher than the physical health component summary, which shows that the patients’ mental health status was better than their physical health.

Bivariate analysis between the demographic and clinical characteristics using the scores of the general symptom distress of SCL-90r and the two summary scores of SF-36 was conducted. Following the bivariate analysis, multivariate linear regression was applied to the independent variables that presented an association of 0.25 (*p* < 0.25). In Table 5, multivariate analysis using multiple linear regression with the stepwise method is presented. According to the multivariate analysis, the existence of psychological distress and expression of mental symptomatology in COVID-19 recovered patients is most frequently predicted by loss of smell, loss of hair and fatigue. In addition, pain, loss of smell, mandatory education, ICU admission, female gender, and the experiencing of skin disturbance are associated with poor physical QoL among COVID-19 recovered patients. The results of multiple linear regression explained 40.1% of the variance. Finally, loss of smell, ICU admission and the experiencing of vision alteration are associated with poor mental QoL among COVID-19 patients. The results of multiple linear regression explained 30.6% of the variance.

## 4. Discussion

The aim of the present study was to investigate perceived symptoms, mental health status and QoL following COVID-19 hospitalization as well as to identify sociodemographic and clinical factors affecting mental health and QoL of COVID-19 recovered patients.

The most frequent symptom reported by the patients during their follow-up was fatigue (67.6%). This result aligns with prior research that identified that the most common post-COVID-19 symptom was observed to be fatigue [20,21]. Post-viral fatigue can occur after almost any viral infection. Nevertheless, it is strongly linked to certain viral diseases including Epstein Barr virus and human immunodeficiency virus. The strong relationship between these viruses and long-term fatigue is assumed to be due to a dysregulated immunological response [22]. According to Townsend et al. (2020), there was no link between COVID-19 severity and the prevalence of persistent fatigue post COVID-19. Female gender and those with a pre-existing diagnosis of depression/anxiety were found to be over-represented in individuals suffering from fatigue in this study [23].

Other symptoms commonly reported by the patients during their follow-up in the present study included physical pain (44.8%), shortness of breath (24.1%), and loss of taste (22.3%), while less frequent symptoms included nausea (3.9%), and nails alteration (3.4%). Similarly, in one study, about half of the participants exhibited post-acute COVID-19 syndrome, with symptoms affecting almost every organ system. Over a 7 to 238-day follow-up period, about three symptoms were reported per patient, and the development of post-acute COVID-19 syndrome was linked substantially with initial COVID-19 severity. The most prevalent symptoms reported by this cohort were cough, tiredness, and dyspnea [24]. Another study found that most COVID-19 patients who required hospitalization still have persisting symptoms even 110 days after release, particularly fatigue and dyspnea, underlining the necessity for long-term follow-up and rehabilitation programs for those patients [25].

In regard to mental health outcomes following COVID-19 hospitalization, it was observed that 11.7% of the recovering patients who were evaluated were experiencing psychological distress. According to SCL-90r, 11.7% of the patients were reporting somatization symptoms, 11.7% obsessive compulsive disorder symptoms, 9.7% depressive symptoms, and 4.8% anger and hostility. The prevalence of psychological distress and psychiatric morbidity among COVID-19 previously hospitalized patients in our study is lower compared with other published studies. According to a cross-sectional study in Cleveland, in which 215 discharged COVID-19 positive patients participated, it was reported that 34%, 24%, and 42% of patients screened positive for PTSD, anxiety, and depression, respectively. Among patients without prior psychiatric history, 42% screened positive for one psychiatric disorder [26]. Similarly, in another quantitative study among COVID-19 discharged patients, high prevalence of psychiatric morbidity was reported. More specifically, the 29.6%, 26.8%, and 25.1% of the total sample screened positive for anxiety, depression, and post-traumatic stress disorder, respectively. Those variations may occur mainly due to the different approach for assessing psychiatric morbidity, as well as due to the self-report measures. Concerning associated factors of psychological distress among those recovered patients, the following were identified in the current study: patients’ loss of smell, loss of hair, and fatigue. These findings are in contrast with findings from previous studies that reported factors such as age, gender, educational status, living with children, death of family member from COVID-19, higher total number of symptoms after discharge, and empiercing discrimination as risk factors for anxiety and depression [27,28].

Regarding QoL, in the present study it was observed that the mean score of both subdomains and summary scores of SF-36 were above 50, meaning that patients reported good QoL in comparison to the general population. This finding contradicts the one in a previous systematic review that showed that regardless of the time since discharge or healing, the post-COVID-19 patients’ quality of life was considerably altered [8]. Our findings can partially be explained by the fact that 88.7% of the participants were living with their spouse or their spouse and children. It is widely accepted and supported by various studies in different populations and diseases that increased family and social support has positive effects in outcomes such as the QoL [29,30]. A person’s ability to fulfill their daily obligations and return to their daily routine and activities demands good physical and mental health. The support that one receives from friends and relatives can have a positive effect on the fulfillment of these efforts. Moreover, the majority of participants in our study had mild to moderate disease, with only a few having severe disease. This could be a contributing cause to the overestimation of QoL.

Perceived symptoms, as well as various clinical and demographic factors, were associated with mental health and QoL of previously hospitalized COVID-19 patients. Specifically, we found that pain, loss of smell, ICU admission, skin disturbances, vision alterations, female gender, and low educational level have a negative impact on both physical and mental summary scores. These findings agree with those of Abdelhafiz et al. 2022 who found that female sex, the existence of comorbidities, lesser education, longer disease duration, as well as severe and critical forms of the disease were all strongly linked with the occurrence of post-COVID symptoms. They also discovered that severe and critical types of the infection, as well as the use of antibiotics and corticosteroids for COVID-19 treatment, were predictors of post-COVID symptoms using regression analysis [31]. Similarly, these findings agree with previous published research, according to which women, critical ill patients, and patients reporting prolonged physical symptoms have poor QoL [6,32,33], and can be interpreted by the fact that QoL is determined by various factors, and not only physical functioning [34].

### Limitations

This study has some limitations. The sample was recruited from one center placed in a peripheral district in Greece, thus results cannot be generalized. Most of the patients suffered from mild to moderate disease and few of them suffered from severe disease. This can be a factor for the overestimation of the QoL. Furthermore, we lack information on symptom history prior to acute COVID-19 illness, as the investigation of the physical and mental health of COVID-19 patients was assessed at one point and also this assessment was based on self-reported instruments. Future studies can focus on various types of health assessment and structured clinical interview at several time points after discharge.

## 5. Conclusions

This study is providing a first glance at physical and mental health after hospitalization on COVID-19 survivors in Greece at their first follow up. Despite the fact that Greek COVID-19 previously hospitalized patients were reporting several symptoms associated with COVID-19, good QoL and mental health were also reported. Further follow-up is needed in order to understand exactly how a person’s level of health changes after this infection and inpatient treatment. In addition, we propose the prospective study of these patients in order to see how the state of health of these individuals evolves over time. Such research is required to protect the physical and mental health and wellbeing of the countless people who survived COVID-19. Our findings can be used to develop effective strategies and early rehabilitation interventions for patients with physical symptoms and mental disorders during the post-hospitalization phase, in order to help them regain as much physical, immune, and mental health as possible. In order to care for this broad and vulnerable population, a multidisciplinary approach is essential.

## Figures and Tables

**Table 1:** Sociodemographic and clinical characteristics.

		*n*	%
Gender	Male	87	60.0
	Female	58	40.0
Age mean ±SD	59.72 ± 12.74		
BMI mean ±SD	29.02 ± 4.42		
Marital Status	Married	114	78.6
	Single	11	7.6
	Cohabitation	2	1.4
	Divorced	3	2.1
	Widowed	15	10.3
Living arrangement	Family	71	49.0
	Alone	17	11.7
	Spouse	39	26.9
	Children	17	11.7
Children mean ±SD	2.06 ± 0.98		
People living together mean ±SD	2.84 ± 1.09		
Occupation	Public servant	26	17.9
	Private employee	23	15.9
	Pension	52	35.9
	Household	15	10.3
	Unemployed	2	1.4
	Other	18	12.4
Education	Primary	41	28.3
	Secondary	56	38.6
	University	33	22.8
	Postgraduate	6	4.1
Area of residence	Urban	85	58.6
	Semiurban	33	22.8
	Rural	27	18.6
Monthly income	Low	22	15.2
	Medium	53	36.6
	High	70	48.3
Multimorbidity	No	82	56.6
	Yes	63	43.4
ICU admission	No	132	91.0
	Yes	13	9
Duration of hospitalization mean ±SD	11.2 ± 8.9		

**Table 2:** Spirometry pulmonary function values.

	Minimum	Maximum	Mean	Std. Deviation
Forced vital capacity (FVC) lit	1.54	6.02	3.40	0.92
Forced vital capacity %	46.70	141.89	94.25	17.49
Forced expiratory volume (FEV1) lit	1.25	5.11	2.76	0.81
Forced expiratory volume %	41.51	133.52	94.79	17.03
FEV1/FVC	54.75	99.51	80.89	6.01
Diffusing capacity of the lungs for carbon monoxide (DLCOc) lit	3.27	11.39	6.60	1.80
Diffusing capacity of the lungs for carbon monoxide %	45.60	112.80	74.89	14.40

**Table 3:** Descriptive statistics of Symptom Checklist 90-R.

Scl-90r Domains	Minimum	Maximum	Mean	Std. Deviation	Presence %
Somatization	0.00	2.67	0.41	0.46	11.7%
Obsessive-compulsive	0.00	1.50	0.41	0.39	11.7%
Interpersonal sensibility	0.00	2.11	0.32	0.34	6.9%
Depression	0.00	2.54	0.48	0.43	9.7%
Anxiety	0.00	1.30	0.27	0.31	2.1%
Anger-hostility	0.00	2.00	0.24	0.34	4.8%
Phobic-anxiety	0.00	1.43	0.11	0.24	3.4%
Paranoid ideation	0.00	2.33	0.29	0.39	4.1%
Psychoticism	0.00	0.60	0.14	0.16	2.1%
General Symptom Index	0.02	16.00	0.44	0.39	11.7%
Positive symptoms	1.00	62.00	16.72	10.38	
Positive symptoms severity	1.00	3.00	1.72	0.47	

**Table 4:** Descriptive statistics for SF-36.

SF-36 QoL	Min	Max	Mean	SD
Physical function (PF)	10.00	100.00	79.96	20.36
Role personal (RP)	0.00	100.00	57.98	42.60
Bodily pain (BP)	0.00	100.00	81.88	26.02
General health (GH)	10.00	100.00	75.95	18.90
Vitality (VT)	10.00	100.00	72.63	19.83
Social functioning (SF)	0.00	100.00	73.61	36.57
Role emotional (RE)	0.00	100.00	77.08	36.23
Mental health (MH)	40.00	100.00	87.51	12.58
Physical component summary (PCS)	7.50	100.00	73.98	20.49
Mental component summary (MCS)	12.50	100.00	77.57	19.03

**Table 5:** Multivariate regression analysis using the stepwise method with dependent variable psychological distress (General Symptom Index of SCL 90-R) and independent variables patient clinical and demographic characteristics (*n* = 145).

Model	Unstandardized Coefficients		Standardized Coefficients	t	Sig.	95.0% Confidence Interval for B	
	B	Std. Error	Beta			Lower Bound	Upper Bound
General Symptom Index of SCL 90-R)							
(Constant)	0.803	0.352		2.281	0.025	0.102	1.505
Loss of smell	1.663	0.528	0.325	3.149	0.002	0.612	2.715
Loss of hairs	1.063	0.437	0.252	2.430	0.017	0.192	1.934
Fatigue	0.999	0.416	−0.250	2.401	0.019	0.170	1.827
F(3.77) = 6.684 *p* = 0.001. R^2^ = 17.6%							
Physical Component Summary (SF-36)							
(Constant)	72.944	5.815		12.545	0.000	61.385	84.503
Pain	−8.540	3.556	−0.209	−2.402	0.018	−15.609	−1.471
Loss of smell	−18.865	4.949	−0.314	−3.812	0.000	−28.703	−9.027
Educational level Mandatory education	0						
Higher education	6.172	2.235	0.238	2.761	0.007	1.729	10.616
ICU admission	−27.011	9.428	−0.237	−2.865	0.005	−45.753	−8.269
Gender male reference category	0						
Female	−8.002	3.454	−0.197	−2.316	0.023	−14.869	−1.135
Skin disturbances	−9.925	4.370	−0.191	−2.272	0.026	−18.612	−1.239
F(3.77) = 11.249 *p* = 0.001. R^2^ = 40.1%							
Mental Component Summary (SF-36)							
(Constant)	84.572	1.774		47.681	0.001	81.047	88.096
Vision alterations	−18.397	3.740	−0.423	−4.919	0.001	−25.829	−10.965
Loss of smell	−19.698	4.656	−0.363	−4.231	0.001	−28.951	−10.445
ICU admission	−19.432	8.455	−0.196	−2.298	0.024	−36.235	−2.628
F(3.77) = 17.005 *p* = 0.001. R^2^ = 30.6%							

## Data Availability

Data supporting this study are available from the corresponding author upon reasonable demand.
